# Evaluation of the upregulation and surface expression of hypoxanthine guanine phosphoribosyltransferase in acute lymphoblastic leukemia and Burkitt’s B cell lymphoma

**DOI:** 10.1186/s12935-020-01457-8

**Published:** 2020-08-06

**Authors:** Michelle H. Townsend, Zac E. Ence, Taylor P. Cox, John E. Lattin, Weston Burrup, Michael K. Boyer, Stephen R. Piccolo, Richard A. Robison, Kim L. O’Neill

**Affiliations:** 1 Department of Microbiology and Molecular Biology, Brigham Young University, Provo, UT USA; 2 Department of Biology, Brigham Young University, Provo, UT USA; 3 Department of Biomedical Informatics, University of Utah, Salt Lake City, UT 84132 USA; 4 Division of Hematology and Hematologic Malignancies, Department of Medicine, Huntsman Cancer Institute, University of Utah, Salt Lake City, UT USA

**Keywords:** Hypoxanthine Guanine Phosphoribosyltransferase (HPRT or HGPRT), Burkitt’s B cell lymphoma, Acute Lymphoblastic Leukemia, ALL, Raji, Lymphocytes, Surface Localization, Biomarker

## Abstract

**Background:**

The aim of this study is to determine whether Hypoxanthine Guanine Phosphoribosyltransferase (HPRT) could be used as a biomarker for the diagnosis and treatment of B cell malignancies. With 4.3% of all new cancers diagnosed as Non-Hodgkin lymphoma, finding new biomarkers for the treatment of B cell cancers is an ongoing pursuit. HPRT is a nucleotide salvage pathway enzyme responsible for the synthesis of guanine and inosine throughout the cell cycle.

**Methods:**

Raji cells were used for this analysis due to their high HPRT internal expression. Internal expression was evaluated utilizing western blotting and RNA sequencing. Surface localization was analyzed using flow cytometry, confocal microscopy, and membrane biotinylation. To determine the source of HPRT surface expression, a CRISPR knockdown of HPRT was generated and confirmed using western blotting. To determine clinical significance, patient blood samples were collected and analyzed for HPRT surface localization.

**Results:**

We found surface localization of HPRT on both Raji cancer cells and in 77% of the malignant ALL samples analyzed and observed no significant expression in healthy cells. Surface expression was confirmed in Raji cells with confocal microscopy, where a direct overlap between HPRT specific antibodies and a membrane-specific dye was observed. HPRT was also detected in biotinylated membranes of Raji cells. Upon HPRT knockdown in Raji cells, we found a significant reduction in surface expression, which shows that the HPRT found on the surface originates from the cells themselves. Finally, we found that cells that had elevated levels of HPRT had a direct correlation to XRCC2, BRCA1, PIK3CA, MSH2, MSH6, WDYHV1, AK7, and BLMH expression and an inverse correlation to PRKD2, PTGS2, TCF7L2, CDH1, IL6R, MC1R, AMPD1, TLR6, and BAK1 expression. Of the 17 genes with significant correlation, 9 are involved in cellular proliferation and DNA synthesis, regulation, and repair.

**Conclusions:**

As a surface biomarker that is found on malignant cells and not on healthy cells, HPRT could be used as a surface antigen for targeted immunotherapy. In addition, the gene correlations show that HPRT may have an additional role in regulation of cancer proliferation that has not been previously discovered.

## Background

Non-Hodgkin lymphomas (NHL) and lymphocytic leukemia (Chronic Lymphoblastic Leukemia and Acute Lymphoblastic Leukemia) are hematological cancers that include more than 30 different cancers of B and T lymphocytes [1]. Non-Hodgkin lymphoma diagnoses made up 4.3% of all new cancer cases in 2017, demonstrating the prevalence of the disease in the United States [2]. In addition, leukemia is the most common malignancy in children, with ALL comprising approximately 26% of all childhood cancers [3, 4].

Cancer biomarkers are typically categorized as diagnostic, prognostic, or predictive. While diagnostic biomarkers identify the onset or presence of cancer, prognostic biomarkers inform physicians of clinical outcomes for their patients throughout treatment, and predictive biomarkers suggest how patients will respond to various treatment regimens[5]. A new category of surface biomarkers has emerged; these biomarkers function as targets for immunotherapy [6–10]. Currently, the most prominent immunotherapy biomarker for B cell malignancies is CD19 [11–15]. CD19 is a type I transmembrane protein expressed in normal and neoplastic B cells, and follicular dendritic cells [16]. CD19 has been used as a direct target for chimeric antigen receptors (CARs) as well as an antibody in bi-specific T-cell that directs cytotoxic T-cells to CD19 expressing B cells [16]. Currently, the only FDA approved CAR therapy targets are against CD19; these include Yescarta (axicabtagene ciloleucel) and Kymriah (Tisagenlecleucel) [17, 18]. A disadvantage of utilizing this biomarker target is that patients’ healthy B cell populations decrease because CD19 is not specific to cancer cells. Another disadvantage of targeting CD19 is that some tumors experience antigen loss which confers resistance to CD-19-targeted immunotherapy, and approximately 10–20% of patients relapse following treatment with CD19-CAR therapy [19, 20]. To aid in reducing antigen loss, researchers seek to identify new immunotherapy biomarkers that can be targeted to eliminate B cell malignancies. New targets such as CD22, CD20, and ROR1 have all shown promise in eliminating certain B cell malignancies, but further research is needed to expand targetable antigens on the surface of malignant cells [21–24].

Previous studies have found that there is variability in regards to hypoxanthine guanine phosphoribosyltransferase (HPRT) expression within malignant tissue [25], and as such it has been suggested that HPRT could be used as targetable biomarker for some solid malignancies [26]. We have designed this study to determine whether HPRT could be used as a targetable biomarker in the treatment of B cell malignancies [25, 27]. In doing this, we hope to identify additional biomarkers options to lessen the growing concern of antigen loss.

## Methods

### Chemicals

Anti-HPRT mouse monoclonal antibody (MA5-15274) used for flow cytometry was aliquoted and stored at − 20 °C (Thermo Fischer Scientific, Waltham, MA, USA). Anti-HPRT rabbit polyclonal antibody (ab10479) used for Western blot analysis were purchased from Abcam (Cambridge, United Kingdom) and stored at 4 °C. Anti-Mouse-FITC and anti-Rabbit-FITC antibodies (Sigma Aldrich, St. Louis, MO, USA) were stored at 4 °C and were used in dark conditions. Goat-anti-rabbit-HRP secondary antibody was purchased from Abcam and stored at 4 °C.

### Cell culture conditions

The Raji (CCL-86- human Burkitt’s B cell lymphoma) cell line was obtained from the American Type Culture Collection (Rockville, MD, USA). NALM-6 cells were gifted by the University of Utah. Raji and NALM-6 cells were grown in RPMI 1640 medium supplemented with 10% fetal bovine serum (FBS) and 2 mM L-Glutamine (all from Hyclone, Logan, UT, USA). Cell media was replaced, and cells were passaged to maintain exponential conditions throughout experimentation. Cell viability was evaluated using trypan blue staining, and cells were utilized for all applications when viability exceeded 98%. All cells were grown at 37 °C and 5% CO_2_. Raji cells were authenticated in May of 2016 by the University of Arizona Genetics Core.

### Flow cytometry

The surface presence of HPRT was evaluated by measuring the fluorescence intensity of anti-HPRT antibodies as previously described [25]. Briefly, all samples were analyzed on a Blue/Red Attune (Applied Biosystems). Using unstained and isotype controls as guides, the positive population was determined by the overall shift in the fluorescent intensity. Each cell line was independently analyzed and the data was plotted using FlowJo Software (FlowJo Enterprise).

### Mononuclear cell separation

Whole blood was collected from healthy volunteers under IRB approval (BYU X090281) with written informed consent. Blood was further diluted with PBS at a 1:1 ratio and layered on top of Lymphocyte Separation Medium (LSM) (Corning Incorporated, Corning, NY, USA) before being centrifuged for 30 min at 400×*g*. The buffy layer was collected and treated with a red blood cell lysis buffer (Biolegend, San Diego, California) before used immediately for experimentation.

### ALL patient samples

Acute lymphoblastic leukemia (ALL) samples were collected at diagnosis or relapse from patients after informed consent utilizing a biobank protocol at the Huntsman Cancer Institute in Salt Lake City, UT. Samples were frozen with dimethyl sulfoxide (DMSO) and albumin and further aliquoted for analysis. Following sufficient thawing at 37 °C, samples were washed with Dulbecco’s phosphate-buffered saline (DPBS). After careful washing, cells were used for flow cytometry analysis and stained with similar procedures as previously described.

### Surface biotinylation and western blot analysis

Cells were analyzed for surface presence of HPRT along with general expression within the cell using the Pierce Cell Surface Protein Isolation Kit (Thermo Scientific, Waltam, MA, USA). Briefly, 3 flasks of Raji cells were grown to 95% confluency and normal lymphocytes were obtained from healthy donors under IRB approval (#090281). Cells were washed and treated with sulfo NHS-SS-biotin. Following rocking on a shaker for 30 min at 4 °C, cells were quenched and treated with a lysis solution for 30 min at 4 °C. Cell lysate was added to a neutravidin gel and incubated for 60 min at room temperature. This solution was run through a filter and washed 4 times. The flow through, containing all unlabeled proteins not found in the plasma membrane, was collected and labeled “unlabeled cellular protein”. The biotin-labelled protein was then eluted from the column utilizing a 50 mM DTT solution and labelled “membrane fraction”. Samples were stored at − 80 °C prior to evaluation with Western Blotting.

Both membrane and unlabeled cellular protein fractions were evaluated for HPRT presence using standard Western Blotting techniques described in Sewda et al. with slight modifications [22]. Briefly, protein was run on a 12% polyacrylamide gel under reducing conditions. Following membrane transfer, membranes were blocked with 5% milk and incubated with HPRT antibodies (1:1000 dilution) and subsequently treated with a Western Bright (Advansta, California, USA) HRP substrate and the image was captured on X-ray film. The film was then analyzed using ImageJ [28] software that calculated the protein expression within each band in reference to the GAPDH control. Samples were run in technical replicates and further confirmed with 3 biological replicates.

### Confocal microscopy

Cells were evaluated qualitatively for HPRT surface localization using procedures previously described [26]. Briefly, cells were labeled with fluorescent antibodies against HPRT and the controls and imaged on a 15 mW Krypton/Argon laser (Bio-Rad Laboratories, Hercules, CA). Cells were not fixed to ensure minimal background fluorescence due to auto-fluorescence. Images were captured and processed using Laser Sharp Computer Software (Bio Rad Laboratories).

### HPRT knockdown

The pSpCas9(BB)-2a- GFP CRISPR vector with an ‘NGG’ protospacer sequence was designed by the Zhang lab [29] and obtained from Addgene (Cambridge, MA, USA).Guide RNA design was conducted using the *CRISPR Design* tool created by MIT [30] with a sequence of “GCTTCATGGCGGCCGTAAAC”. Briefly, Raji cells were grown to a concentration of 4 × 10^5^ cells per mL and seeded in a 6-well plate. Following 24 h of growth, cells were transfected with a lipofectamine LTX reagent (Invitrogen Waltam, MA, USA). Briefly, 150 µl of Opti-MEM (Gibco, Gaithersburg, MD) was incubated with 5–7 µl of lipofectamine LTX reagent while 250 µl of Opti-MEM was incubated with approximately 2 × 10^3^ng of the CRISPR vector. The solutions were mixed together and incubated at room temperature for 30 min. The lipofectamine-DNA solution was then added to the Raji cells in a drop-wise fashion. Cells were grown for 3 days and then treated with media containing 6-thioguanine (6-TG) at a final concentration of 10 µg/µL. 6-TG is a nucleoside analog that is toxic to cells with a functional HPRT gene. Cells that survived the 6-TG treatment were grown to sufficient quantities to produce cell extract. This extract was analyzed by Western blotting using similar techniques described previously to confirm surviving cells were HPRT^−/−^. The final cell population was labeled “knockdown” to account for the incomplete knockout of HPRT in all cells. As the cell population did not result from a single clone, there were some HPRT expressing cells within the population after selection.

### Gene expression analysis of malignant B cell lines and patient samples

We evaluated gene-expression levels for 105 genes across 79 malignant human B cell lines from the Broad Institute’s Cancer Cell Line Encyclopedia[31]. The genes chosen for this analysis were based on their association with cancer development and progression. Several sources were used to determine optimal genes of interest [32–43], and genes chosen were not strictly limited to blood cancers. Of the genes associated with cancer development, selections were made to include proteins involved in immunity, tumor suppression, metastasis, drug resistance, and general development. We used RNA-Sequencing data for protein-coding transcripts that had been generated using Illumina-based, short-read sequencing. These data had been processed using the kallisto software [44], then log- transformed and converted to transcripts-per- million values [45]. This data can be found at https://osf.io/gqrz9/files/ (matrices/CCLE/CCLE_tpm.tsv.gz). We summed the transcript-level values to gene-level values and sorted the cell lines according to HPRT1 expression level, from high to low expression per sample. We parsed and prepared the data using Python (https://python.org, v.3.6.1) scripts. In making the heat map, we used the R (v.3.4.3) statistical package [46] and the Superheat package (v.0.1.0) [47].

### Gene-expression analysis of adult B-acute lymphoblastic leukemia

We obtained gene-expression data for 191 patients who had been diagnosed with adult B-lineage.

Acute lymphoblastic leukemia. These data had been generated using NimbleGen Human.

Expression Arrays (which use 60mer probes). We obtained these data from Gene Expression.

Omnibus (GSE34861) in preprocessed form. We used the R statistical package (v.3.4.3) to plot.

these data.

### Statistical analysis

ANOVA statistical analysis with the Tukey-Kramer multiple comparison method were used to analyze the flow cytometry data from all cell lines, representing the differential surface expression of HPRT for the various treatments. In addition, two-way ANOVA tests were performed to compare the mean expression of HPRT between Raji^WT^ and knockdown cells. All statistical analyses were performed using GraphPad Prism 7 software. Differences were considered significant when a *p value* was < 0.05.

When assessing relationships between HPRT expression and other genes, we used a Spearman correlation test to calculate correlation coefficients and two-sided p-values. In performing these calculations, we used the cor.test function in the stats package of the R (v.3.4.3) statistical software.

## Results

### Raji cells show a significant increase in HPRT localization on the plasma membrane while healthy cells have insignificant expression

**Fig. 1:**
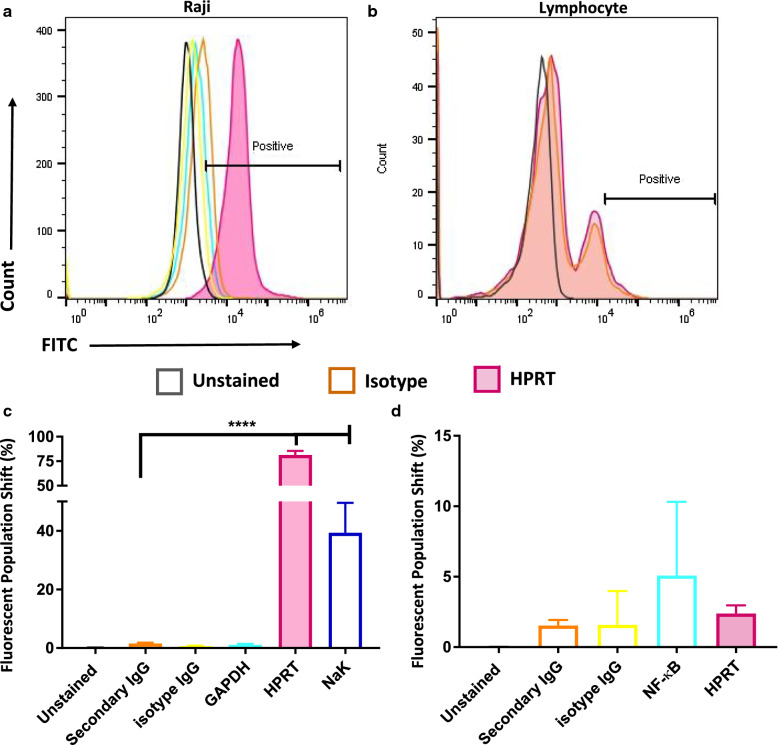
HPRT surface localization in Raji and normal cells. Fluorescent HPRT antibodies were compared against isotype controls to determine increase in HPRT expression on the surface of both Raji and normal lymphocytes. GAPDH and NF-kB were used as additional negative controls to ensure internal proteins were not stained and NaK was used as a positive membrane protein control. **a** Raji cells stained with a fluorescent anti-HPRT antibody experienced a significant shift (p-value < 0.0001) when compared to isotype controls. **b** Normal lymphocytes from healthy donors stained with fluorescent anti-HPRT antibodies did not experience a significant shift in the fluorescent population when compared to isotype controls. **c** Statistical analysis reveals a significant elevation of HPRT expression on the surface of Raji cells, and **d** an insignificant elevation of HPRT on healthy lymphocytes

Raji cells labeled with antibodies against HPRT had an average fluorescent population shift of 81.39% which was significantly different (p-value < 0.0001) from the isotype controls, which only experienced a 1.50% shift in the fluorescent population (Fig. 1a, c). Lymphocytes from healthy donors labeled with antibodies against HPRT had insignificant fluorescent shifts in the population (1.53%) when compared to isotype controls (*p-value* = 0.98) (Fig. 1b, d). These results indicate that HPRT has substantial presence on the surface of Raji cells and has insignificant presence on the surface of their normal counterparts.

**Fig. 2:**
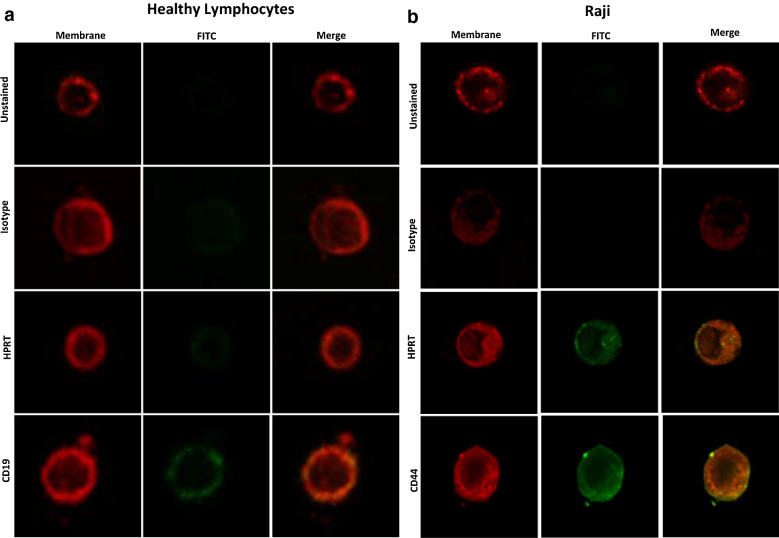
HPRT expressed highly on the plasma membrane of Raji cells. Fluorescent HPRT antibodies were compared against a membrane specific dye to highlight overlap in binding. CD19 and CD44 were used as positive controls and isotype controls were used as negative controls to highlight nonspecific antibody binding. **a** Healthy lymphocytes did not have a significant presence of HPRT on the cell surface and levels were similar to isotype controls. **b** Raji cells showed a clear increase in fluorescence when analyzed for HPRT and there was a direct overlap between the membrane dye and the antibody treatment, indicating that HPRT is co-localized with the plasma membrane of Raji cells

To confirm surface localization, malignant and normal cells were analyzed using confocal microscopy to visualize direct overlap between the plasma membrane and HPRT binding. Raji cells had a direct overlap between the membrane specific dye and the FITC conjugated HPRT antibody resulting in a yellow merged image (Fig. 2b). This same overlap was not observed in normal lymphocytes as the HPRT binding was similar to that of the isotype control, showing that these cells had minimal HPRT expression (Fig. 2a). This analysis shows that HPRT associates strongly with the plasma membrane and has a significant surface presence on malignant Raji cells.

### Biotinylated surface proteins show HPRT bound to the plasma membrane of Raji cells

**Fig. 3:**
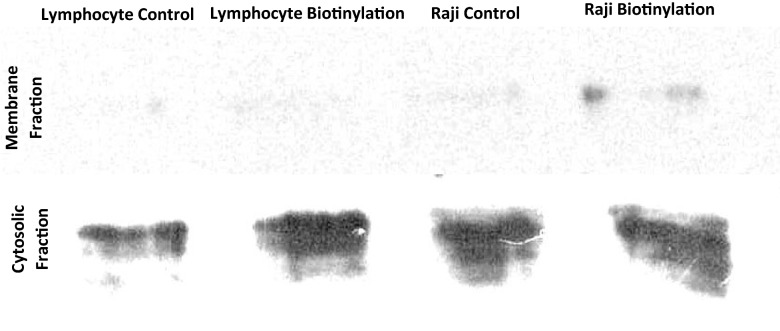
Biotinylated surface proteins reveal HPRT presence and confirms surface presence of the protein. ‘Membrane Fraction’ shows the total surface proteins on both lymphocytes and Raji samples. ‘Cytosolic Fraction’ shows the total HPRT within the cell. A band is observed in the ‘Raji Biotinylation’ sample as the membrane fraction of Raji cells and healthy lymphocytes are probed for HPRT presence

To further confirm whether HPRT was bound to the plasma membrane of Raji cells, we biotinylated the surface proteins of Raji cells and normal cells, and probed for HPRT presence. This analysis revealed a band in the Raji membrane biotin sample that was absent from the normal lymphocyte membrane biotin sample and all other membrane controls. As expected, the band observed in the membrane fraction was smaller than that of the unlabeled cellular protein fraction as the amount of HPRT on the cell surface would be significantly less than the internal levels of the protein (Fig. 3). This analysis further confirmed the localization of HPRT on the cell surface of Raji cells and the absence of the enzyme on normal cells.

### HPRT knockdown cells exhibited reduced levels of surface HPRT expression

**Fig. 4:**
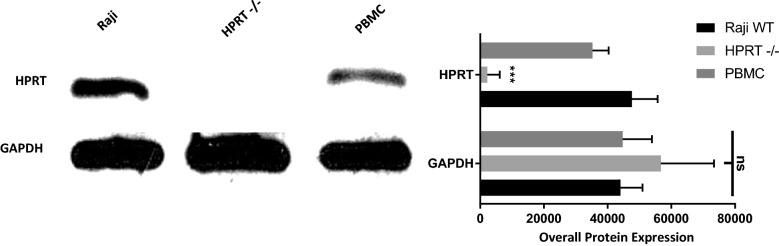
HPRT knockdown confirmation. Following knockdown of HPRT, a western blot was performed to both confirm knockdown status and to also quantify the internal cellular expression of HPRT within Raji cells and healthy PBMC. Knockdown cells had significantly decreased levels of HPRT in total cell lysate, indicating successful knockdown (*p-value* = 0.0002). Healthy PBMCs had significantly lower total HPRT than Raji samples

To help confirm that the surface HPRT originated from the cells themselves, we created a knockdown of HPRT in Raji cells using a CRISPR system. Following adequate selection, we determined that there was sufficient reduction of HPRT within the cells for analysis (Fig. 4). The average relative expression of the enzyme went from 47,628 in wild type Raji cells to 2254 in knockdown cells (*p-value* = 0.0002).

**Fig. 5:**
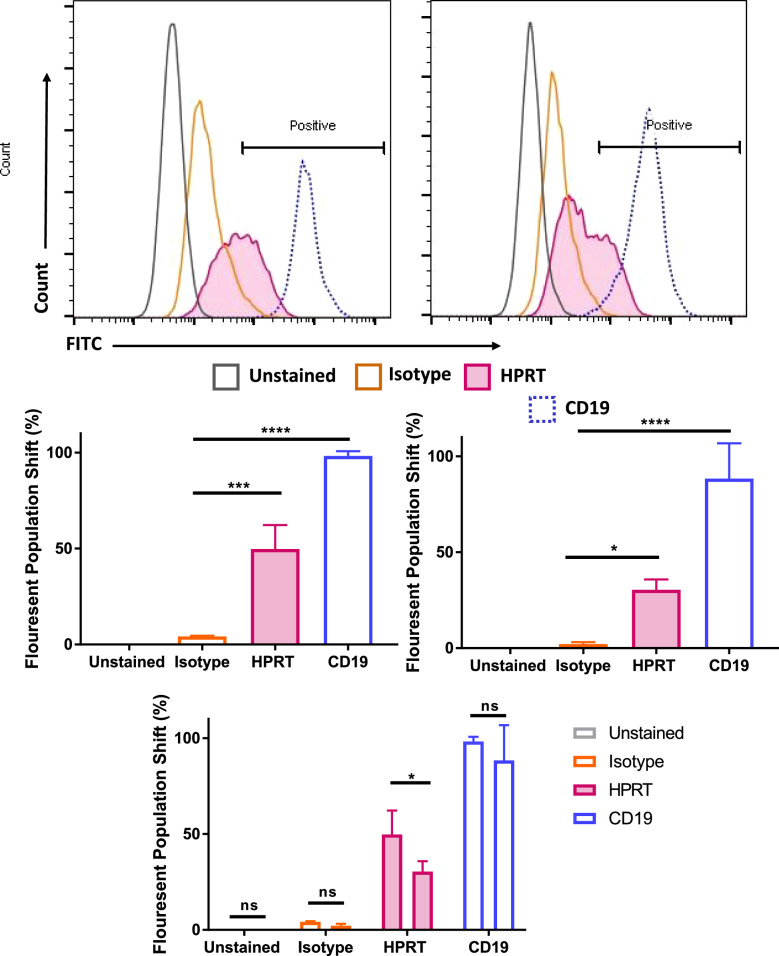
Flow analysis of HPRT knockdown Raji cells reveal a reduction in surface binding. Following knockdown of the HPRT gene in Raji cells, we analyzed surface HPRT expression in both knockdown Raji and wild type Raji cells. We found that there was a significant decrease in HPRT surface localization in the knockdown when compared to the wild type Raji cells

When evaluating HPRT knockdown cells for surface expression we found a significant (*p-value* = 0.039) decrease in the presence of the protein on the surface compared to the WT Raji counterparts (Fig. 5). We observed a shoulder in the population that we hypothesize are a result of the sample not being a true knockout, but a knockdown. While the knockdown cells did show slight significance in expression when compared to isotype controls (*p-value* = 0.029), this was far less than the surface expression of HPRT in WT Raji cells (*p-value* = 0.0001). The overall average reduction in HPRT expression upon protein knockdown was approximately 20%. Further analysis with a true knockdown cell line will need to be evaluated to confirm these initial findings, but these data indicate that surface HPRT is directly produced within the cells.

### Analysis of patient samples shows that HPRT surface expression has clinical relevance

**Fig. 6:**
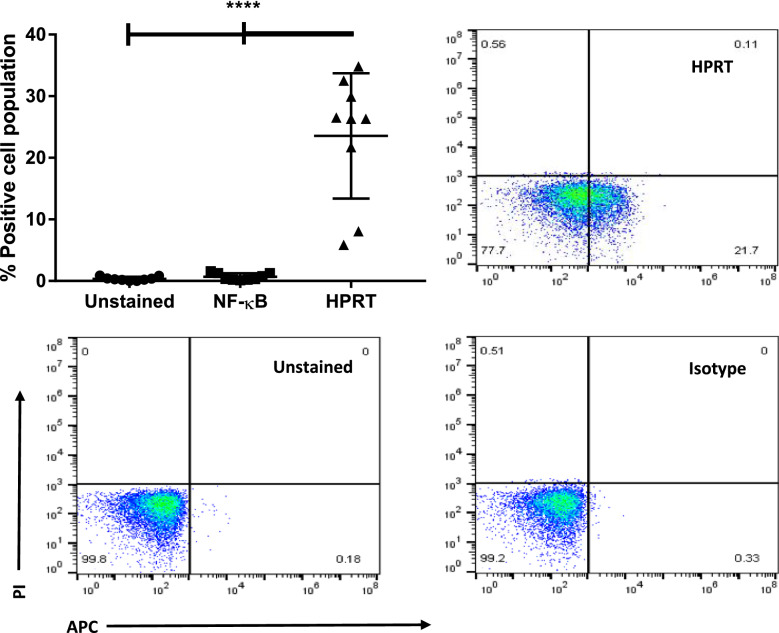
ALL patients show elevated surface HPRT. Patient samples were stained with PI to discriminate against dead cells. APC conjugated secondary antibodies were used in conjunction with protein-specific primary antibodies to stain proteins of interest. Upon evaluation of 9 ALL patient samples, we found that 7 of them had elevated HPRT surface localization with an average fluorescent population shift of 25%. This indicates that the surface localization observed in Raji cells is also found within patients

To determine whether the presence of HPRT was an artifact of cell culturing conditions or cell immortalization, we analyzed samples from patients with ALL to determine whether the phenomenon was also found within these patients. We found that 7 out of the 9 patient samples were positive for elevated HPRT on the cell’s surface and we saw an overall increase in fluorescence (*p-value* < 0.0001) upon anti-HPRT staining when compared to isotype controls. Samples with the highest fraction of HPRT positive cells observed had approximately 34% positives, while the lowest had 6.7%, with the average positive fraction around 25% for ALL patients (Fig. 6). This analysis showed that HPRT has relevance within a proportion of patients. This analysis also confirmed that HPRT surface localization is not a universal characteristic of malignant cells and patients should be evaluated on an individual basis.

### Differential gene expression between HPRT low and HPRT high expressing cancer cells

**Fig. 7:**
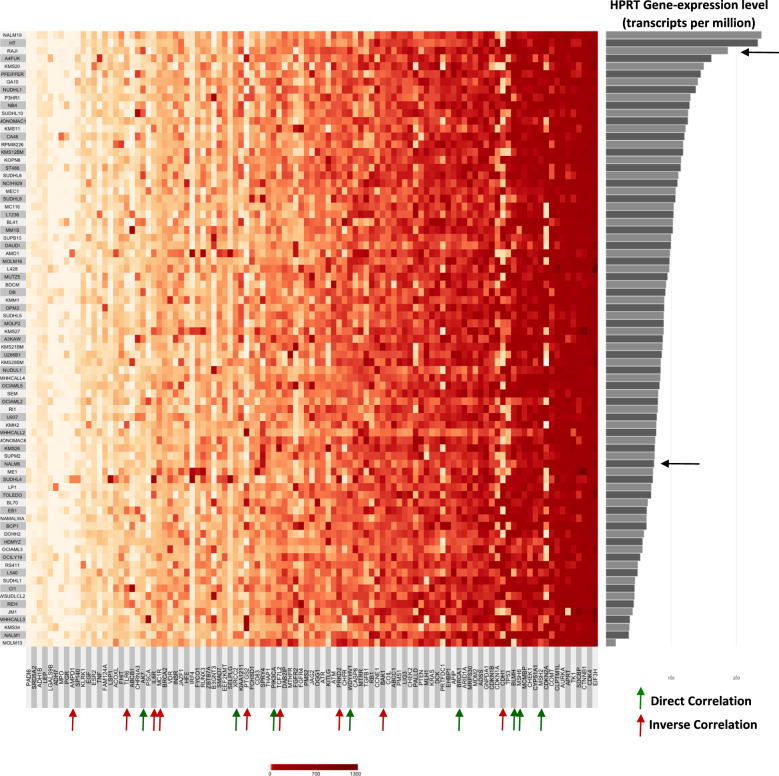
Gene-expression evaluation of HPRT high vs. HPRT low expression B cell lines. 79 Cancerous B cell lines are ranked on the Y-axis according to their relative HPRT expression, which is portrayed on the righthand Y-axis. The expression of 105 cancer-associated genes are labeled on the X-axis. The expression of each of these genes is portrayed with higher expression scaled to darker color. We found significant variability within B cell lines in terms of HPRT expression and also identified correlative relationships between the gene expression of HPRT and other cancer-associated genes

As we found variability between normal cells and malignant cells in regard to their relative HPRT expression, we further evaluated changes in gene expression between high-expressing and low-expressing cells to determine whether HPRT could have any potential influence on other cancer-associated genes. We assessed 79 different malignant B cell lines and ranked them according to their relative HPRT expression. Raji cells had the third highest expression of all cell lines evaluated, which we predicted, as there is significant surface presentation of the enzyme in Raji cells (Fig. 7).

Many other genes experienced a significant trend correlating to HPRT expression (Table 1). Genes that showed a direct positive correlation to HPRT were XRCC2 (*p-value* = 0.0045), BRCA1 (*p-value* = 0.0032), PIK3CA *(p-value* = 0.0034), MSH2 (*p-value* = 0.0445), MSH6 (*p-value* = 0.019), WDYHV1 (*p-value* = 0.0066), AK7 (*p-value* = 0.0452), and BLMH (*p-value* = 0.0498). Genes that showed an inverse correlative relationship to HPRT were PRKD2 (*p-value* = 0.0109), PTGS2 (*p-value* = 0.0046), TCF7L2 (*p-value* = 0.0032), CDH1 (*p-value* = 0.0201), IL6R (*p-value* = 0.0054), MC1R (*p-value* = 0.0487), AMPD1 (*p-value* = 0.0227), TLR6 (*p-value* = 0.0401), and BAK1 (*p-value* = 0.0052). Although HPRT is not the sole contributor to these changes in gene expression, there may be a cascading relationship between HPRT levels and these genes as there are general trends either towards higher expression or lower expression when HPRT is elevated within the cells.

**Table 1:** HPRT gene correlations to 109 genes in 79 human malignant B cell lines in the CCLE dataset

Gene name	Gene	General function	p-value
Direct correlation			
	XRCC2	DNA repair protein involved in homologous recombination.	0.0045
Breast cancer type 1 susceptibility protein	BRCA1	Tumor suppressor gene that maintains genomic stability via DNA damage repair, chromatin remodeling, transcriptional regulation and apoptosis.	0.0032
Phosphatidylinositol 4,5-bisphosphate 3-kinase catalytic subunit alpha	PIK3CA	Involved in cell growth, survival, proliferation, motility and morphology. Also participates in cellular signaling in response to growth factors.	0.0034
	MSH2	Involved in mismatch repair system.	0.0445
	MSH6	Involved in mismatch repair system.	0.019
Protein N-terminal glutamine amidohydrolase	WDYHV1	Involved in the N-end rule pathway in protein degradation.	0.0066
Adenylate kinase 7	AK7	Nucleoside monophosphate kinase that transfers phosphate groups between nucleoside triphosphates and monophosphates.	0.0452
Bleomycin hydrolase	BLMH	Cysteine peptidase, hydrolyzes homocysteine thiolactone	0.0598
Inverse correlation			
Serine/threonine-protein kinase D2	PRKD2	Regulation of cell proliferation via MAP1/3 signaling.	0.0109
Prostaglandin G/H synthase 2	PTGS2	Production of inflammatory prostaglandins	0.0046
	TCF7L2	Involved in the Wnt signaling pathway and modulates MYC expression.	0.0032
Cadherin-1	CDH1	Involved in mechanisms regulating cell-cell adhesion, mobility, and proliferation.	0.0201
Interleukin-6 receptor	IL6R	Potent pleiotropic pro-inflammatory cytokine that regulates cell growth and differentiation.	0.0054
Melanocyte-stimulating hormone receptor	MC1R	Produces melanin pigment	0.0487
AMP deaminase 1	AMPD1	Energy metabolism	0.0227
Toll-like receptor 6	TLR6	Innate immune response to Gram-positive bacteria and fungi	0.0401
Brassinosteroid insensitive 1-associated receptor kinase 1	BAK1	Controls the expression of genes associated with innate immunity in the absence of pathogens or elicitors.	0.0052

### Low expressing B cell line shows no significant surface localization of HPRT

**Fig. 8:**
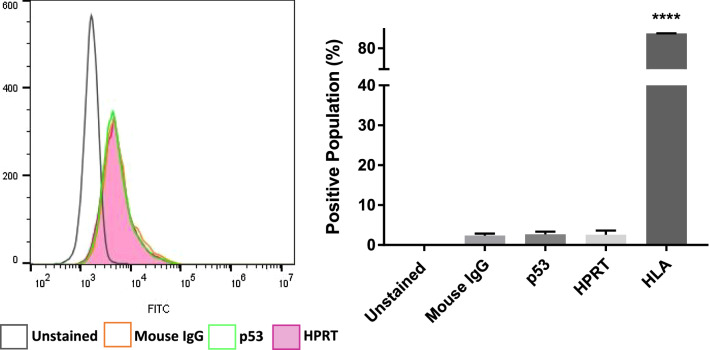
Expression of HPRT in NALM-6 cells. NALM-6 cells expressed lower amounts of HPRT internally and we evaluated whether that impacted the overall surface expression of the protein. HLA antibodies were utilized as positive controls while p53 and isotype antibodies were used as negative controls for statistical analysis. We found that there was no significant surface localization of HPRT in NALM-6 cells

In order to determine whether the elevation of HPRT within the cell lines was contributing to the observed surface expression, we evaluated the surface localization of HPRT in Nalm-6 cells which have relatively low overall expression. In these cells we found that there was insignificant surface localization of HPRT (*p-value* = 0.995) when compared to isotype controls (Fig. 8). This would indicate that the surface expression of the protein is most likely linked to the elevated internal expression of the protein.

### ALL patients experience a similar diversity of HPRT expression as cell lines

**Fig. 9:**
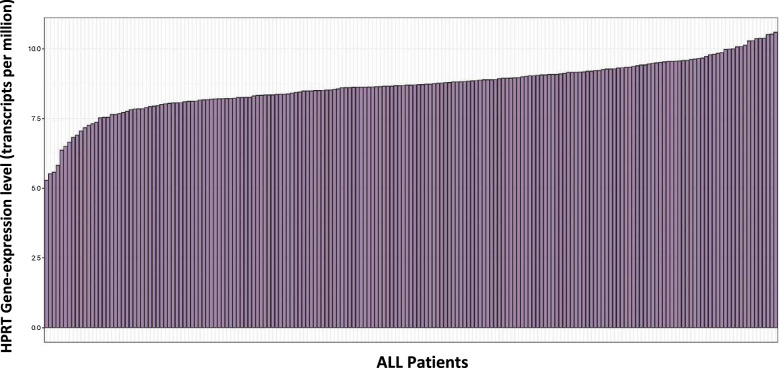
HPRT internal cellular expression of HPRT in ALL patient samples. We evaluated the overall expression of HPRT in ALL patients to determine whether there was similar variability in expression. We found that while some patients exhibited a high expression of HPRT, some patients showed much lower levels

As we saw a variety of different surface expression profiles in patient samples, we evaluated the distribution of overall HPRT expression within patients. We found that there was a similar spread of HPRT elevation within ALL patients with the highest expressing patients showing levels above 10x of the lower expressing patients according to mRNA levels (Fig. 9). This data along with previous data supports the hypothesis that HPRT surface expression is impacted by elevated overall internal mRNA and protein expression and we hypothesize that the patients who experienced an insignificant surface localization of the protein had a lower overall expression profile compared to those that had higher levels.

## Discussion

HPRT is an enzyme that plays a critical role in the cell cycle by providing essential nucleotides that support cell division and DNA replication. We have shown that HPRT is significantly elevated in some patient malignancies. This elevation appears to manifest via co-localization to the plasma membrane of the cell. Yet, this surface expression is not found on all malignant cells and we have shown that many cell lines have significant variation in regard to their expression of HPRT. As cell cycle regulation is a common target for mutation in malignant cells, we hypothesize that enzymes controlling the cell cycle are the most likely contributing factor to the differential HPRT expression within these cells [48, 49]. Additionally, we hypothesize that surface presence of the enzyme is related to an overabundance of the protein internally. This suggests, that cell lines with an unusually high level of HPRT will have significant surface expression of the protein. HPRT has been known to be found in vesicles transporting nucleosides to the plasma membrane and functions to take up hypoxanthine. In cells deficient in HPRT, hypoxanthine accumulates in the extracellular medium while the ribose moiety of inosine remains in the cell. Therefore, HPRT plays a critical role in nucleoside transport. Due to this role, HPRT has shown a relationship in mammalian cells to the plasma membrane, and has a 10-fold greater tendency to remain associated with the plasma membrane in mouse L929 cells when compared to adenosine kinase [50]. Because nucleoside transporters are required for translocation of nucleosides between intracellular compartments, they are found in the plasma membrane of most cell types. Therefore, as cancer cells are known to abundantly secrete extracellular vesicles [51], it is possible that the increase in vesicle transportation and nucleoside uptake could result in HPRT membrane localization in these cells. Additionally, as it has been shown that other human cancer cell lines have shown no apparent upregulation of the protein on the surface, we do not believe HPRT is associating with the membrane due to its release from dying cells [52] otherwise it would be a universal phenomenon.

HPRT could be used as a cancer-associated epitope for immunotherapy targeting as its expression is limited to malignant cells. New epitopes are required as cancer is an evolving disease and adapts to avoid immune detection [9]. There has been unprecedented success using CD19 Chimeric Antigen Receptors to target and kill malignant B cells [11–13, 16, 53]. Yet, this therapy is not cancer-specific and targets healthy cells as well. As a protein that appears to be found only on malignant cells, HPRT could serve as a safer target for patients with B cell malignancies, as they may maintain their healthy supply of B cells. HPRT could also serve as a novel biomarker to aid in increasing numbers of CD19-resistent cancers [19, 54]. Targeting HPRT could serve as an additional companion target to possibly treat cells that become resistant to current treatment regimes.

While HPRT is present on the surface of Raji cells, we hypothesize that only cells with significantly elevated HPRT production express the enzyme on the plasma membrane. Screening patients for surface HPRT would be feasible; a simple blood test would confirm whether a patient was positive or negative. Our data indicates that HPRT surface localization is a relatively common occurrence in these B cell malignancies and could be a valuable biomarker in future therapeutic treatments or as a companion biomarker for cancer cell identification and classification. HPRT would not be suitable for use as a single diagnostic due to the variable nature of its expression between patients, but if a symptomatic patient presented with elevated HPRT it might suggest the presence of an underlying B cell malignancy, warranting further evaluation. Thus, HPRT might be a useful screening biomarker to identify patients who might have an underlying B-cell malignancy. Future work will need to be conducted using a larger number of patient samples to determine whether targeting HPRT would be technically feasible and beneficial from a therapeutic standpoint.

While the surface expression of HPRT may be useful as a biomarker for diagnosis and treatment, novel correlations between HPRT and other genes may highlight possible regulatory roles that HPRT plays within the cell. Of the 17 genes that had a significant correlation to HPRT expression, 9 are involved in cellular proliferation and DNA synthesis/repair. With this is mind, HPRT may be responsible for additional regulation of cellular proliferation outside of nucleotide synthesis and may interact or direct other genes. Another possibility is that the same genes that are regulating cellular proliferation in these genes may also influence HPRT expression. On an interesting note, of the 9 genes with an inverse correlative relationship with HPRT expression, 4 genes (PTGS2, IL-6R, TLR6, and BAK1) are involved in the regulation and activation of the immune system. This may suggest that the upregulation of HPRT could have a side effect of downregulating the immune system. Of special interest is the inverse correlation HPRT expression has with IL-6R, which is known to play an important role in B cell growth. IL-6 induces B cell proliferation by binding to receptor complexes and activates the Jak/STAT signaling. Within cancerous cells IL-6 is often upregulated in the serum of patients and can contribute to disease stage and shorter survival rates [55]. The exact role between HPRT and IL-6 is not known and we speculate that the relationship between the two is found in the role HPRT plays in GTP production as Jak/STAT signaling relies on GTPases [56]. Yet, this relationship needs further investigation to determine how the two proteins are related.

In addition, we also noted some interesting cell lines that have gene profiles significantly different from any other cell line. SUDHL4, AMO1, and L428 cells appear to have inverse gene expression to the average B cell line. This highlights that any observed correlations between gene expression are the result of several different contributing factors, and not just HPRT expression within these cells.

## Conclusions

Because HPRT is localized to the surface of malignant lymphocytes, it has the potential to be used as a targetable biomarker for immunotherapy. As antigen escape is emerging as a significant concern with targeted immunotherapy, the need to find and use new biomarkers is always increasing. In addition, the genes that are correlated with HPRT expression may elucidate a new role of HPRT in cancer proliferation.

## Data Availability

The datasets supporting the conclusions of this study and are included in this article. Any request for data or material can be sent to the corresponding author.

## Abbreviations

HPRT: Hypoxanthine guanine phosphoribosyltransferase
ALL: Acute lymphoblastic leukemia
CLL: Chronic lymphoblastic leukemia
BRCA1: Breast cancer type 1 susceptibility protein
PIK3CA: Phosphatidylinositol 4,5-bisphosphate 3-kinase catalytic subunit alpha
WDYHV1: Protein N-terminal glutamine amidohydrolase
AK7: Adenylate kinase 7
BLMH: Bleomycin hydrolase
PRKD2: Serine/threonine-protein kinase D2
PTGS2: Prostaglandin G/H synthase 2
CDH1: Cadherin-1
IL6R: Interleukin-6 receptor subunit alpha
MC1R: Melanocyte-stimulating hormone receptor
AMPD1: AMP deaminase 1
TLR6: Toll-like receptor 6
BAK1: Brassinosteroid insensitive 1-associated receptor kinase 1

## References

[1] Epidemiology in B-cell malignancies [Internet]. http://www.targetedonc.com/publications/special-reports/2014/hematologic-malignancies-issue1/epidemiology-in-b-cell-malignancies. Accessed 10 Jan 2018.

[2] Non-hodgkin lymphoma. Cancer Stat Facts [Internet]. https://seer.cancer.gov/statfacts/html/nhl.html. Accessed 10 Jan 2018.

[3] Key Statistics for Non-Hodgkin Lymphoma in Children [Internet]. https://www.cancer.org/cancer/childhood-non-hodgkin-lymphoma/about/key-statistics.html. Accessed 10 Jan 2018.

[4] Cancers that Develop in Children [Internet]. https://www.cancer.org/cancer/cancer-in-children/types-of-childhood-cancers.html. Accessed 10 Jan 2018.

[5] Due H, Svendsen P, Bødker JS, Schmitz A, Bøgsted M, Johnsen HE, et al. miR-155 as a biomarker in B-cell malignancies. Biomed Res Int [Internet]. Hindawi; 2016;2016:1–14. http://www.hindawi.com/journals/bmri/2016/9513037/. Accessed 10 Jan 2018. 10.1155/2016/9513037 PMC4884835 27294145

[6] Aggen DH, Drake CG. Biomarkers for immunotherapy in bladder cancer: a moving target. J Immunother Cancer [Internet]. BioMed Central; 2017;5:94. https://jitc.biomedcentral.com/articles/10.1186/s40425-017-0299-1. Accessed 10 Jan 2018. 10.1186/s40425-017-0299-1 PMC5697433 29157296

[7] Rodriguez-Vida A, Strijbos M, Hutson T (2016). Predictive and prognostic biomarkers of targeted agents and modern immunotherapy in renal cell carcinoma. ESMO open.

[8] Gulley JL, Berzofsky JA, Butler MO, Cesano A, Fox BA, Gnjatic S, et al. Immunotherapy biomarkers 2016: overcoming the barriers. J Immunother Cancer [Internet]. BioMed Central; 2017;5:29. http://jitc.biomedcentral.com/articles/10.1186/s40425-017-0225-6. Accessed 10 Jan 2018. 10.1186/s40425-017-0225-6 PMC5359902 28653584

[9] Yuan J, Hegde PS, Clynes R, Foukas PG, Harari A, Kleen TO (2016). Novel technologies and emerging biomarkers for personalized cancer immunotherapy. J Immunother cancer.

[10] Schumacher TN, Kesmir C, van Buuren MM (2015). Biomarkers in cancer immunotherapy. Cancer Cell.

[11] Tasian SK, Gardner RA (2015). CD19-redirected chimeric antigen receptor-modified T cells: a promising immunotherapy for children and adults with B-cell acute lymphoblastic leukemia (ALL). Therapeutic Adv Hematol.

[12] Lorentzen CL, Straten PT (2015). CD19-chimeric antigen receptor T cells for treatment of chronic lymphocytic leukemia and acute lymphoblastic leukemia. Scand J Immunol.

[13] Maude SL, Teachey DT, Porter DL, Grupp SA (2016). CD19-targeted chimeric antigen receptor T-cell therapy for acute lymphoblastic leukemia. Blood.

[14] Long AH, Haso WM, Shern JF, Wanhainen KM, Murgai M, Ingaramo M (2015). 4-1BB costimulation ameliorates T cell exhaustion induced by tonic signaling of chimeric antigen receptors. Nat Med.

[15] Davila ML, Brentjens RJ (2016). CD19-Targeted CAR T cells as novel cancer immunotherapy for relapsed or refractory B-cell acute lymphoblastic leukemia. Clin Adv Hematol Oncol.

[16] Wang K, Wei G, Liu D. CD19: a biomarker for B cell development, lymphoma diagnosis and therapy. Exp Hematol Oncol [Internet]. BioMed Central; 2012;1:36. http://ehoonline.biomedcentral.com/articles/10.1186/2162-3619-1-36. Accessed 10 Jan 2018. 10.1186/2162-3619-1-36 PMC3520838 23210908

[17] Alegre MM, Robison RA, Neill KLO (2013). Thymidine kinase 1: a universal marker for cancer. Cancer Clin Oncol.

[18] Maude SL, Laetsch TW, Buechner J, Rives S, Boyer M, Bittencourt H (2018). Tisagenlecleucel in children and young adults with B-cell lymphoblastic leukemia. N Engl J Med.

[19] Fischer J, Paret C, El Malki K, Alt F, Wingerter A, Neu MA (2017). CD19 isoforms enabling resistance to CART-19 immunotherapy are expressed in B-ALL patients at initial diagnosis. J Immunother.

[20] Sharma P, Hu-Lieskovan S, Wargo JA, Ribas A (2017). Leading edge review primary, adaptive, and acquired resistance to cancer immunotherapy. Cell.

[21] Fry TJ, Shah NN, Orentas RJ, Stetler-Stevenson M, Yuan CM, Ramakrishna S (2017). CD22-targeted CAR T cells induce remission in B-ALL that is naive or resistant to CD19-targeted CAR immunotherapy. Nat Med.

[22] Haso W, Lee DW, Shah NN, Stetler-Stevenson M, Yuan CM, Pastan IH (2013). Anti-CD22-chimeric antigen receptors targeting B-cell precursor acute lymphoblastic leukemia. Blood.

[23] Till BG, Jensen MC, Wang J, Qian X, Gopal AK, Maloney DG (2012). CD20-specific adoptive immunotherapy for lymphoma using a chimeric antigen receptor with both CD28 and 4–1BB domains: pilot clinical trial results. Blood.

[24] Hudecek M, Schmitt TM, Baskar S, Lupo-Stanghellini MT, Nishida T, Yamamoto TN (2010). The B-cell tumor-associated antigen ROR1 can be targeted with T cells modified to express a ROR1-specific chimeric antigen receptor. Blood.

[25] Townsend MH, Felsted AM, Ence ZE, Piccolo SR, Robison RA, O’Neill KL. Elevated expression of hypoxanthine guanine phosphoribosyltransferase within malignant tissue. Cancer Clin Oncol [Internet]. 2017;6:19. http://ccsenet.org/journal/index.php/cco/article/view/70556.

[26] Townsend MH, Anderson MD, Weagel EG, Velazquez EJ, Weber KS, Robison RA (2017). Non-small-cell lung cancer cell lines A549 and NCI-H460 express hypoxanthine guanine phosphoribosyltransferase on the plasma membrane. Onco Targets Ther.

[27] Monnat RJ, Chiaverotti T, Hackmann a F, Maresh G (1992). a. Molecular structure and genetic stability of human hypoxanthine phosphoribosyltransferase (HPRT) gene duplications. Genomics.

[28] Schindelin J, Rueden CT, Hiner MC, Eliceiri KW (2015). The ImageJ ecosystem: An open platform for biomedical image analysis. Mol Reprod Dev.

[29] Ran FA, Hsu PD, Wright J, Agarwala V, Scott DA, Zhang F (2013). Genome engineering using the CRISPR-Cas9 system. Nat Protoc.

[30] Optimized CRISPR. Design [Internet]. http://crispr.mit.edu/. Accessed 22 Mar 2018.

[31] Barretina J, Caponigro G, Stransky N, Venkatesan K, Margolin AA, Kim S (2012). The Cancer Cell Line Encyclopedia enables predictive modelling of anticancer drug sensitivity. Nature.

[32] Waters CE, Saldivar JC, Hosseini SA, Huebner K. The FHIT gene product: tumor suppressor and genome “caretaker” [Internet]. Cell. Mol. Life Sci. Birkhauser Verlag AG; 2014;4577–87. /pmc/articles/PMC4233150/?report = abstract. Accessed 17 July 2020. 10.1007/s00018-014-1722-0 PMC4233150 25283145

[33] Snook AE, Eisenlohr LC, Rothstein JL, Waldman SA (2007). Cancer mucosa antigens as a novel immunotherapeutic class of tumor-associated antigen. Clin Pharmacol Ther.

[34] Park DJ, Lesueur F, Nguyen-Dumont T, Pertesi M, Odefrey F, Hammet F, et al. Rare mutations in XRCC2 increase the risk of breast cancer. Am J Hum Genet [Internet] Elsevier. 2012;90:734–9. /pmc/articles/PMC3322233/?report = abstract. Accessed 17 July 2020. 10.1016/j.ajhg.2012.02.027 PMC3322233 22464251

[35] Baba Y, Nosho K, Shima K, Irahara N, Kure S, Toyoda S, et al. Aurora—a expression is independently associated with chromosomal instability in colorectal cancer. neoplasia [Internet]. 2009;11:418–25. http://linkinghub.elsevier.com/retrieve/pii/S147655860980050X. 10.1593/neo.09154 PMC2671854 19412426

[36] Abbas T, Dutta A. P21 in cancer: intricate networks and multiple activities [Internet]. Nat. Rev. Cancer. Nature Publishing Group; 2009;400–14. /pmc/articles/PMC2722839/?report = abstract. Accessed 17 July 2020. 10.1038/nrc2657 PMC2722839 19440234

[37] He L, Shen Y. Mthfr C677T polymorphism and breast, ovarian cancer risk: a meta-analysis of 19,260 patients and 26,364 controls. Onco Targets Ther [Internet]. Dove Medical Press Ltd.; 2017;10:227–38. /pmc/articles/PMC5229257/?report = abstract. Accessed 17 July 2020. 10.2147/OTT.S121472 PMC5229257 28123304

[38] Park J, Chen L, Tockman MS, Elahi A, Lazarus P (2004). The human 8-oxoguanine DNA N-glycosylase 1 (hOGG1) DNA repair enzyme and its association with lung cancer risk. Pharmacogenetics.

[39] Chen F, Liu X, Bai J, Pei D, Zheng J. The emerging role of RUNX3 in cancer metastasis (Review). Oncol Rep. Spandidos Publications; 2016;1227–36. https://pubmed.ncbi.nlm.nih.gov/26708741/. Accessed 17 July 2020. 10.3892/or.2015.4515 26708741

[40] Tang A, Gao K, Chu L, Zhang R, Yang J, Zheng J. Aurora kinases: novel therapy targets in cancers [Internet]. Oncotarget. Impact Journals LLC; 2017;23937–54. /pmc/articles/PMC5410356/?report = abstract. Accessed 17 July 2020. 10.18632/oncotarget.14893 PMC5410356 28147341

[41] Liu XS, Haines JE, Mehanna EK, Genet MD, Ben-Sahra I, Asara JM, et al. ZBTB7A acts as a tumor suppressor through the transcriptional repression of glycolysis. Genes Dev [Internet]. Cold Spring Harbor Laboratory Press; 2014;28:1917–28. /pmc/articles/PMC4197949/?report = abstract. Accessed 17 July 2020. 10.1101/gad.245910.114 PMC4197949 25184678

[42] Hou H, Sun D, Zhang X. The role of MDM2 amplification and overexpression in therapeutic resistance of malignant tumors [Internet]. Cancer Cell Int. BioMed Central Ltd.; 2019;216. https://cancerci.biomedcentral.com/articles/10.1186/s12935-019-0937-4. Accessed 17 July 2020. 10.1186/s12935-019-0937-4 PMC6704499 31440117

[43] Ni Z, Tao K, Chen G, Chen Q, Tang J, Luo X, et al. CLPTM1L is overexpressed in lung cancer and associated with apoptosis. PLoS ONE [Internet]. Public Library of Science; 2012;7:52598. /pmc/articles/PMC3530437/?report = abstract. Accessed 17 July 2020. 10.1371/journal.pone.0052598 PMC3530437 23300716

[44] Bray NL, Pimentel H, Melsted P, Pachter L (2016). Near-optimal probabilistic RNA-seq quantification. Nat Biotechnol.

[45] Tatlow P, Piccolo SR. A cloud-based workflow to quantify transcript-expression levels in public cancer compendia. Sci Rep [Internet]. Nature Publishing Group; 2016;6:39259. http://www.nature.com/articles/srep39259. 10.1038/srep39259 PMC5159871 27982081

[46] TEAM RDC. Statutes of “ The R Foundation for Statistical Computing ” means to meet the objectives. 2005;1–5.

[47] Barter RL, Yu B. Superheat: An R package for creating beautiful and extendable heatmaps for visualizing complex data. 2015. http://arxiv.org/abs/1512.01524. 10.1080/10618600.2018.1473780 PMC6430237 30911216

[48] Maddika S, Ande SR, Panigrahi S, Paranjothy T, Weglarczyk K, Zuse A (2007). Cell survival, cell death and cell cycle pathways are interconnected: implications for cancer therapy. Drug Resist Updat.

[49] Evan GI, Vousden KH. Proliferation, cell cycle and apoptosis in cancer. Nature. 2001;411. 10.1038/35077213 11357141

[50] Li C-C, Hochstadt J. Membrane-associated enzymes involved in nucleoside processing by plasma membrane vesicles isolated from L,, cells grown in defined medium* [Internet]. http://www.jbc.org/. 814124

[51] Rahbarghazi R, Jabbari N, Sani NA, Asghari R, Salimi L, Kalashani SA, et al. Tumor-derived extracellular vesicles: reliable tools for cancer diagnosis and clinical applications. Cell Commun. Signal. BioMed Central Ltd.; 2019. 10.1186/s12964-019-0390-y PMC6617682 31291956

[52] Townsend MH, Felsted AM, Cox TP, Ence ZE, Piccolo SR, Robison RA, et al. Abstract 562: HPRT surface localization on prostate cancer cells as a biomarker for immunotherapy. Cancer Res. American Association for Cancer Research (AACR); 2018. p. 562–562.

[53] Lorentzen CL, Straten PT, Ctx PEN. CD19-chimeric antigen receptor T cells for treatment of chronic lymphocytic leukaemia and acute lymphoblastic leukaemia. 2015. 10.1111/sji.12331 26099639

[54] Ruella M, Maus MV (2016). Catch me if you can: leukemia escape after CD19-directed T cell immunotherapies. Comput Struct Biotechnol J.

[55] Burger R. Impact of interleukin-6 in hematological malignancies. Transfus Med Hemotherapy 2013;40:336 10.1159/000354194 PMC3822278 24273487

[56] Pelletier S, Duhamel F, Coulombe P, Popoff M, Meloch S. Rho family GTPases are required for activation of Jak/STAT signaling by G protein-coupled receptors—PubMed—NCBI [Internet]. Mol Cell Biol. 2003. p. 1316–33. https://www.ncbi.nlm.nih.gov/pubmed/12556491. Accessed 4 Oct 2018. 10.1128/MCB.23.4.1316-1333.2003 PMC141129 12556491

